# Supplementary material for: Near-infrared to ultra-violet frequency conversion in chalcogenide metasurfaces
Source: Nat Commun. 2021 Oct 5;12:5833. doi: 10.1038/s41467-021-26094-1 (PMC8492647; doi:10.1038/s41467-021-26094-1)
Supplement: Supplementary file 1 — Supplementary Information [file 41467_2021_26094_MOESM1_ESM.pdf]

Supplementary Information

Near-Infrared to Ultra-Violet Frequency Conversion in Chalcogenide Metasurfaces

Jiannan Gao,[1] Maria Antonietta Vincenti,[2] Jesse Frantz,[3] Anthony Clabeau,[4] Xingdu Qiao,[5] Liang Feng,[6] Michael Scalora,[7] Natalia M. Litchinitser[1*]

[1]Department of Electrical and Computer Engineering, Duke University, Durham, NC, 27708, USA
[2]Department of Information Engineering – University of Brescia, Via Branze 38, 25123 Brescia, Italy
[3]US Naval Research Laboratory, 4555 Overlook Ave., SW, Washington, DC 20375, USA
[4] University Research Foundation, 6411 Ivy Ln. 110, Greenbelt, MD 20770, USA
[5]Department of Electrical and Systems Engineering, University of Pennsylvania, Philadelphia, PA 19104, USA
[6]Department of Materials Science and Engineering, University of Pennsylvania, Philadelphia, PA 19104, USA
[7]Aviation and Missile Center, US Army CCDC, Redstone Arsenal, AL 35898-5000 USA
* *natalia.litchinitser@duke.edu*

Supplementary Note 1

Theoretical model

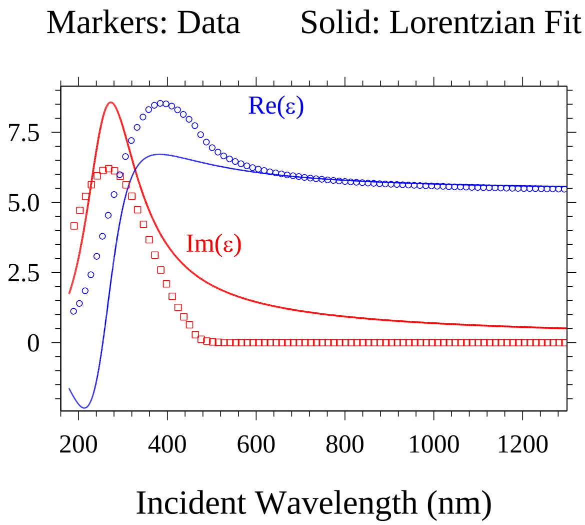

Supplementary Figure 1. The linear dielectric constant of our sample with Lorentzian function fitting.

The linear dielectric constant of our sample is reported in Supplementary Figure 1

(markers). Also in Supplementary Figure 1 a single Lorentzian function suffices to approximate the dielectric response. It is worthy of note that the material is opaque below 500nm. The material equation of motion is derived and described in details elsewhere and is as follows:

$$\ddot{\mathbf{P}}_b + \tilde{\gamma}_b \dot{\mathbf{P}}_b + \tilde{\omega}_{0,b}^2 \mathbf{P}_b - \tilde{\beta}(\mathbf{P}_b \bullet \mathbf{P}_b)\mathbf{P}_b = \frac{n_{0,b} e^2 \lambda_0^2}{m_b^* c^2}\mathbf{E} + \frac{e\,\lambda_0}{m_b^* c^2}(\mathbf{P}_b \bullet \nabla)\mathbf{E} + \frac{e\,\lambda_0}{m_b^* c^2}\dot{\mathbf{P}}_b \times \mathbf{H} \; . \; (1)$$

$\mathbf{P}_b$ is the polarization of the bound electrons. In other words, Supplementary Equation (1) describes the behavior of electrons that are not allowed to leave atomic sites. Since chalcogenides are centrosymmetric, the second order bulk nonlinearity is neglected. Free charges play no role and their effect is also neglected. The nonlinear, third-order response is assumed to be isotropic, and is taken into account by the term $\mathbf{P}_b = -\tilde{\beta}(\mathbf{P}_b \bullet \mathbf{P}_b)\mathbf{P}_b$. The parameter $\tilde{\beta} \approx \omega_{0,b}^2 \lambda_r^2 / \left(L^2 n_{0b}^2 e^2 c^2\right)$ is a scaled coefficient that may be derived from a nonlinear oscillator model. $\omega_{0,b}$ is the resonance frequency, $n_{0b} = 10^{22}/\mathrm{cm}^3$ is the bound electron density, $\lambda_r = 1\mu\mathrm{m}$ is a convenient reference wavelength, c is the speed of light in vacuum, $L = 3\times 10^{-8}\,\mathrm{cm}$ is the lattice constant. The resonance is located between 200 nm and 300 nm, so that $\tilde{\beta} \approx 10^{-8}$. This parameter thus determines nonlinear dispersion as exemplified by the complex functions $\chi_{\omega}^{(3)}$ and $\chi_{3\omega}^{(3)}$, which describe all third order effects triggered by bound electrons, including self-phase modulation, nonlinear absorption, and THG conversion efficiencies. The lattice constant L represents the maximum allowed extension of a classical spring, which for solids can vary between 1Å and 5Å, a disparity that is reflected in the particle density and that may substantially affect the magnitude of $\tilde{\beta}$. With this in mind, both $\chi_{\omega}^{(3)}$ and $\chi_{3\omega}^{(3)}$ may be derived from Supplementary Equation (1), and the result is plotted in Supplementary Figure 2. Therefore, integration of Supplementary Equation (1) together with Maxwell's equation also keeps track of both linear and nonlinear dispersions.

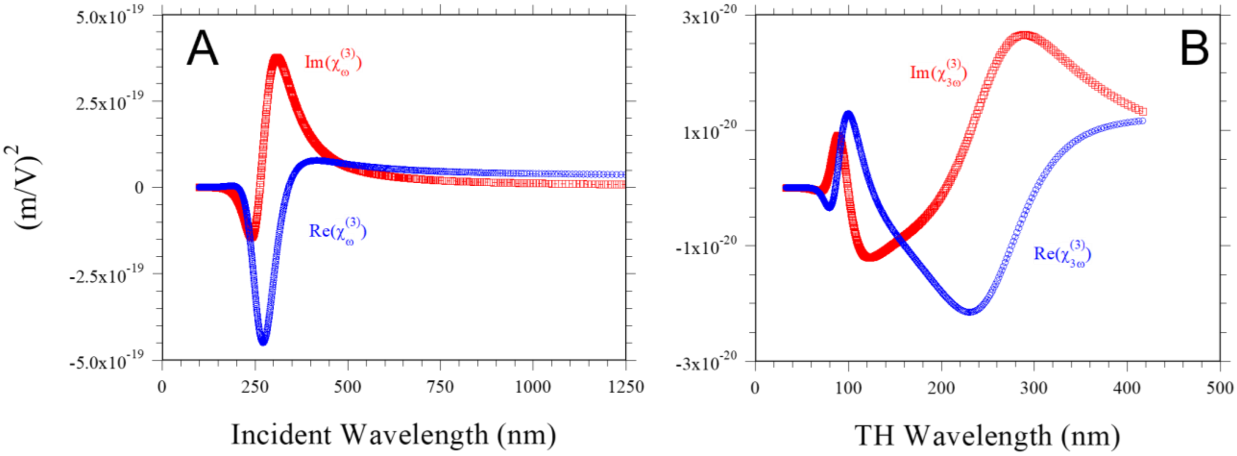

Supplementary Figure 2. The nonlinear dispersion derived from Supplementary Equation (1). A. The nonlinear dispersion of fundamental wavelength. **B.** The nonlinear dispersion of TH.

The use of supplementary equation (1) allows us to describe the process by expanding the fields near the surface, and by considering the consequences of terms like $\frac{e\,\lambda_0}{m_0^* c^2}\left(\mathbf{P}_{bj}\bullet\nabla\right)\mathbf{E}$, which represents surface nonlinearities, and the magnetic Lorentz contribution $\frac{e\,\lambda_0}{m_{bj}^* c^2}\dot{\mathbf{P}}_{bj}\times\mathbf{H}$, which contains both surface and volume nonlinear bound currents. Both terms are fully expanded up to their third harmonic contributions, allowing for pump depletion and down-conversion to occur. These terms account for SHG in centrosymmetric materials.

Supplementary Note 2

Linear and nonlinear characterization

Here we show additional linear (Supplementary Figure 3) and nonlinear (Supplementary Figure 4) measurements resultscompared numerical simulations.

Supplementary Figure 4A shows the experimental nonlinear measurements, while Supplementary Figure 4B reports the theoretical enhancement expected for the TH signal from

the metasurface with respect to TH signal from the unpatterned As_2S_3 layer. The peak of the TH enhancement in panel B corresponds to a conversion efficiency of ~2.1×10^{-8}.

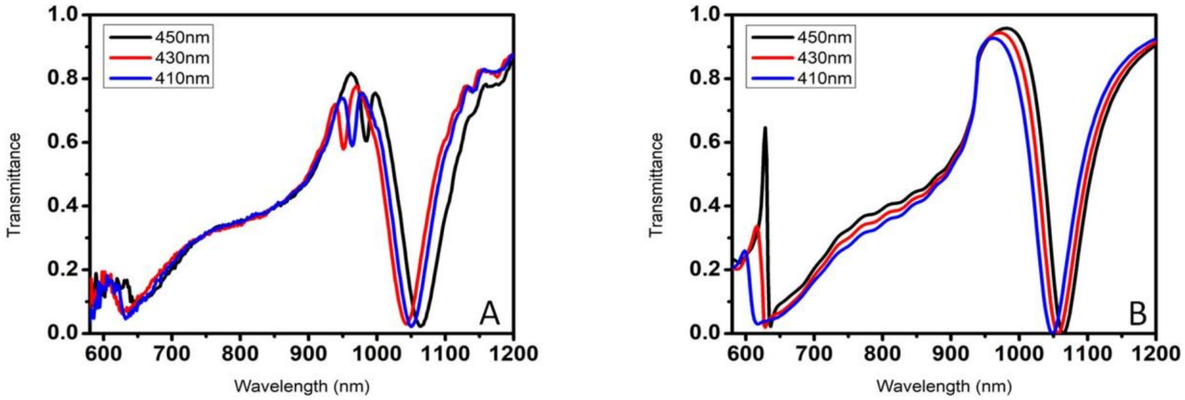

Supplementary Figure 3. Comparison of measured (A) and calculated (B) linear transmission for a range of metasurface unit cell sizes.

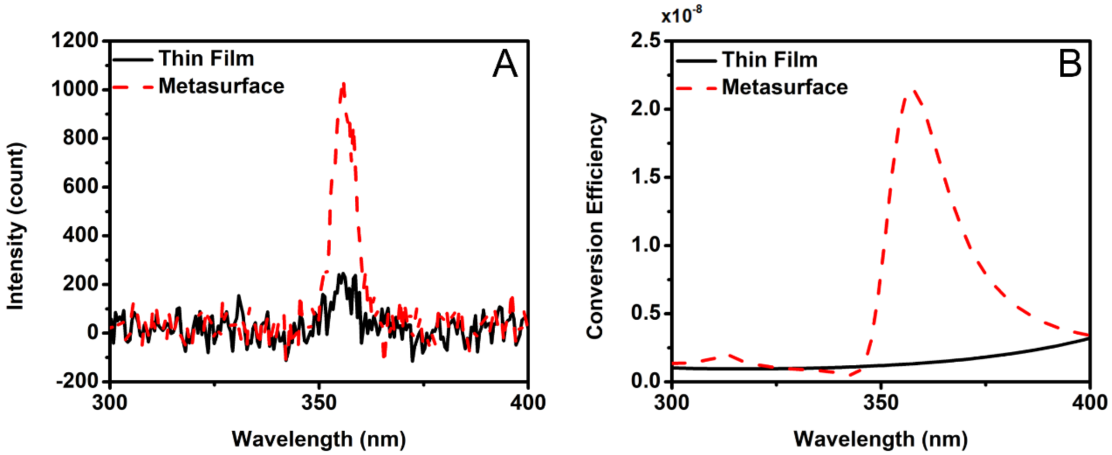

Supplementary Figure 4. Comparison of measured (A) and calculated (B) nonlinear field enhancement.

Supplementary Figure 5 shows numerical simulations of the electric and magnetic field enhancement inside the unit cell of the metasurface. Field enhancements for both fields are calculated with respect to the incident fields (E_0 and H_0).

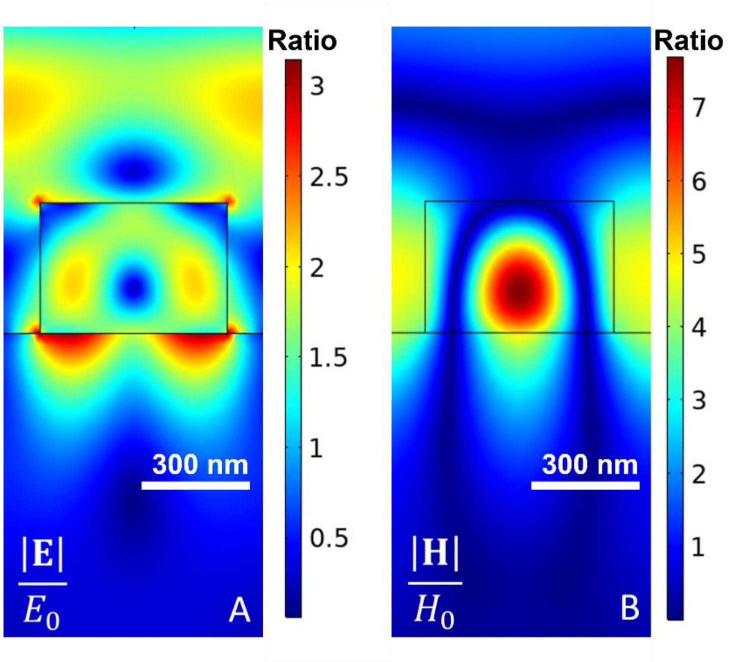

Supplementary Figure 5. Electric (A) and magnetic (B) field enhancement at resonance (1052nm) for nanowire size w_x = 430 nm. Numerical simulations confirm significant field enhancement for both electric and magnetic fields inside the nanosctructure. The spatial decoupling of the fields suggests energy velocities of order c/100.
